# An Integrative Analysis of Identified Schizophrenia-Associated Brain Cell Types and Gene Expression Changes

**DOI:** 10.3390/ijms231911581

**Published:** 2022-09-30

**Authors:** Wenxiang Cai, Weichen Song, Zhe Liu, Dhruba Tara Maharjan, Jisheng Liang, Guan Ning Lin

**Affiliations:** 1 Shanghai Mental Health Center, Shanghai Jiao Tong University School of Medicine, School of Biomedical Engineering, Shanghai Jiao Tong University, Shanghai 200030, China; 2 Shanghai Key Laboratory of Psychotic Disorders, Shanghai 200030, China

**Keywords:** schizophrenia, cell types proportions, differential expression genes, functional pathways, CIBERSORTx

## Abstract

Schizophrenia (SCZ) is a severe mental disorder that may result in hallucinations, delusions, and extremely disordered thinking. How each cell type in the brain contributes to SCZ occurrence is still unclear. Here, we leveraged the human dorsolateral prefrontal cortex bulk RNA-seq data, then used the RNA-seq deconvolution algorithm CIBERSORTx to generate SCZ brain single-cell RNA-seq data for a comprehensive analysis to understand SCZ-associated brain cell types and gene expression changes. Firstly, we observed that the proportions of brain cell types in SCZ differed from normal samples. Among these cell types, astrocyte, pericyte, and PAX6 cells were found to have a higher proportion in SCZ patients (astrocyte: SCZ = 0.163, control = 0.145, P.adj = 4.9 × 10^−4^, effect size = 0.478; pericyte: SCZ = 0.057, control = 0.066, P.adj = 1.1 × 10^−4^, effect size = 0.519; PAX6: SCZ = 0.014, control = 0.011, P.adj = 0.014, effect size = 0.377), while the L5/6_IT_CAR3 cells and LAMP5 cells are the exact opposite (L5/6_IT_Car3: SCZ = 0.102, control = 0.108, P.adj = 0.016, effect size = 0.369; LAMP5: SCZ = 0.057, control = 0.066, P.adj = 2.2 × 10^−6^, effect size = 0.617). Next, we investigated gene expression in cell types and functional pathways in SCZ. We observed chemical synaptic transmission dysregulation in two types of GABAergic neurons (PVALB and LAMP5), and immune reaction involvement in GABAergic neurons (SST) and non-neuronal cell types (endothelial and oligodendrocyte). Furthermore, we observed that some differential expression genes from bulk RNA-seq displayed cell-type-specific abnormalities in the expression of molecules in SCZ. Finally, the cell types with the SCZ-related transcriptomic changes could be considered to belong to the same module since we observed two major similar coordinated transcriptomic changes across these cell types. Together, our results offer novel insights into cellular heterogeneity and the molecular mechanisms underlying SCZ.

## 1. Introduction

Schizophrenia (SCZ) is a neuropsychiatric disorder characterized by disorganized speech, auditory hallucinations, and cognitive impairment [1]. SCZ affects approximately 1% of the worldwide population [2]. Most SCZ patients are affected by genetic and early environmental risk factors that disrupt brain development, particularly in some neuronal subtypes and brain regions [3].

Many efforts have already been carried out to investigate the pathology of the disease. Some studies show that the dorsolateral prefrontal cortex (DLPFC) is a crucial region associated with cognitive deficits and working memory [4,5]. Abnormal bioenergetic pathways have been reported in schizophrenia in the DLPFC, including a decrease in the expression of genes encoding proteins involving the malate shuttle, tricarboxylic acid (TCA) cycle, and ubiquitin metabolism [6]. Moreover, impaired neuronal signaling in the DLPFC can influence synchronized patterns of neural activity that ultimately influence cognition and behavior [7]. However, brain cell types have not been thoroughly studied in SCZ, and not all brain cell types would be similarly affected in SCZ. It is essential to identify the contribution of each cell type to SCZ. For instance, GABAergic neurons provide both inhibitory and disinhibitory modulation of cortical and hippocampal circuits, and it was reported that these functions are altered in schizophrenic (SCZ) subjects [8,9,10]. Astrocytes can critically affect key neurodevelopmental and homeostatic processes of schizophrenia pathogenesis, including glutamatergic signaling, synaptogenesis, synaptic pruning, and myelination [11,12]. In addition, a common-variant genome-wide association results for SCZ from a mouse map to a limited set of brain cells such as pyramidal cells, medium spiny neurons (MSNs), and certain interneurons, but far less consistently to the embryonic, progenitor, or glial cells [13].

Meanwhile, using multi-omics integrative analysis to explore the pathogenesis of SCZ has already resulted in hundreds of genetic loci being identified to associate with SCZ [14]. The studies above provided some information about SCZ-related cell types and potential critical genetic loci, but they focused only on a single cell type in the brain or used omics data from mouse models instead of human patients. These results are not comprehensive enough to confirm and decipher the genetic and molecular mechanisms of SCZ. Thus, it is important to apply single-cell RNA sequencing (scRNA-seq) on human SCZ samples, which allows us to explore cell composition and cell-type-specific molecular alterations at the single-cell level.

Limited by the lack of single-cell RNA-seq data from human SCZ patients, we used CIBERSORTx [15], a tool that can estimate cell type abundances from bulk tissue transcriptomes, infers cell-type-specific gene expression profiles, and allows the use of single-cell RNA-sequencing data without single-cell sorting.

In this study, we provided large-scale changes in the neuronal and non-neuronal transcriptomes of SCZ patients using CIBERSORTx. In particular, we showed that some cell types exhibited cell proportion changes and a dramatic SCZ-related cell-special dysregulation of gene expression. Next, by performing gene ontology analysis on the DEGs of each cell type, we observed cell-type-special function pathways underlying SCZ. Moreover, we identified ten genes that overlapped between bulk RNA-seq and scRNA-seq DEGs and their cell-type-special changes pattern. In addition, we estimated the Jaccard similarity score among each cell type to identify which cell types belong to the same module. Thus, our results indicate that the SCZ-related cell types and cell-type-special gene expression changes could help understand and study the potential pathogenic mechanism in SCZ.

## 2. Results

### 2.1. Identification of Changes in Cell-Type Proportion Using Signature Matrix

**Figure 1:**
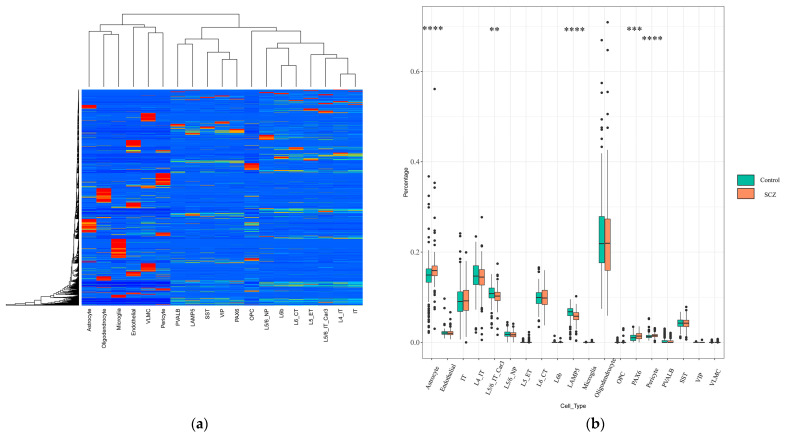
Distribution of brain cell types in bulk RNA-seq data. (**a**) Signature gene matrix of inferred 19 brain cell types by study. Heatmap showing a custom signature matrix created from scRNA-seq data with CIBERSORTx; (**b**) box plots depict distributions of 19 brain neuronal and non-neuronal cell types between SCZ and normal samples (depicted *p*-values are from the Wilcoxon test). ** *p* < 0.01; *** *p* < 0.001; **** *p* < 0.0001. OPC, oligodendrocyte precursor cell; VLMC, vascular leptomeningeal cell; IT, intratelencephalic; NP, near-projecting; CT, corticothalamic; ET, extratelencephalic–pyramidal tract.

We downloaded 6468 cell samples of single-cell RNA-seq data [16,17] clustered from 19 neuronal and non-neuronal cell types isolated from middle temporal gyrus (MTG) and anterior cingulate cortex (ACC) tissues from normal adults. The 19 cell types included L4_ IT, L5_ET, L5/6_NP, L5/6_IT_CAR3, L6_CT, L6b, IT, LAMP5, SST, VIP, PAX6, PVALB, astrocyte, endothelial, microglia, oligodendrocyte, OPC, pericyte, and VLMC. Based on the CIBERSORTx algorithm, a signature matrix including genes of the 19 cell types was created (Figure 1a; Table 1). We also downloaded RNA-seq data of schizophrenia patients and normal controls from BrainSeq Phase 2 [18].

Next, to identify the proportion change of each cell type of bulk samples, applying the CIBERSORTx algorithm to RNA-seq data with the signature matrix obtained above, we estimated the relative proportions of 19 neuron and non-neuron cell subsets of the DLPFC between SCZ patients and normal samples (Figure 1b). Within all brain cell types, astrocyte, pericyte, and PAX6 cells were found to have a higher proportion in SCZ patients than in normal people (astrocyte: SCZ = 0.163, control = 0.145, P.adj = 4.9 × 10^−4^, effect size = 0.478; pericyte: SCZ = 0.057, control = 0.066, P.adj = 1.1 × 10^−4^, effect size = 0.519; PAX6: SCZ = 0.014, control = 0.011, P.adj = 0.014, effect size = 0.377). In addition, we observed a notable decrease in the proportion of several cell types, such as the L5/6_IT_CAR3 cell type and LAMP5 cell type (L5/6_IT_Car3: SCZ = 0.102, control = 0.108, P.adj = 0.016, effect size = 0.369; LAMP5: SCZ = 0.057, control = 0.066, P.adj = 2.2 × 10^−6^, effect size = 0.617). The rest of the cell types had no significant differences between SCZ patients and normal controls.

### 2.2. Bulk RNA-Seq Revealed Abnormal Gene Expression and Immune-Related Pathways in SCZ Samples

**Figure 2:**
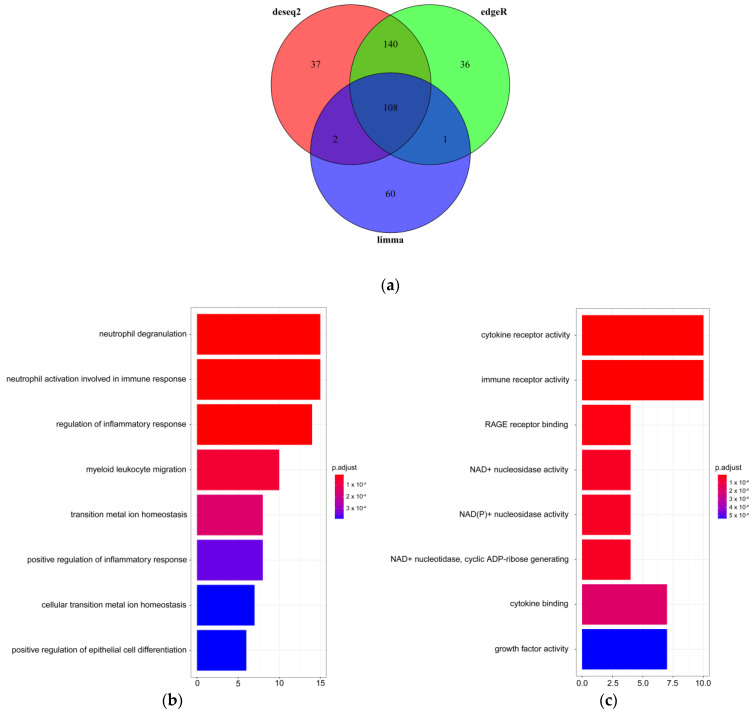
DEGs and related pathways of bulk RNA-seq. (**a**) Venn diagram of DEGs by three differential expression algorithms (DEseq2, LIMMA, and edgeR); (**b**,**c**) The top significantly overrepresented GO BP and MF terms. The x-axis represents the count of genes. The y-axis indicates the items of GO BP. The color represents the value of the FDR-adjusted *p*-value.

To identify gene expression change and whether these DEGs enriched functional pathways associated with SCZ, we first used the bulk RNA-seq data for differential analysis. Taking the overlapped results from differential expression genes (DEGs) from three R packages “DESeq-2” [19], “limma” [20], and “edgeR” [21], 108 DEGs (77 down-regulated and 31 up-regulated) were detected. (Figure 2a). Next, we applied gene enrichment (GO) analysis to the DEGs. We observed that the enrichment of DEGs was associated with gene ontology biological processes (BP) terms related to neutrophil degranulation, neutrophil activation involved in immune response, regulation of inflammatory response, and so on (Figure 2b). In addition, the enrichment of DEGs was associated with gene ontology molecular function (MF) terms related to functions, such as cytokine receptor activity, immune receptor activity, and NAD^+^ nucleosidase activity (Figure 2c). The analysis showed that DEGs of bulk RNA-seq converged to immune-related pathways, further indicating that immune response played a significant role in SCZ pathogenesis.

### 2.3. PVALB, SST, and LAMP5 Neurons Involved in Chemical Synaptic Transmission and Immune Function Dysregulation in SCZ

**Figure 3:**
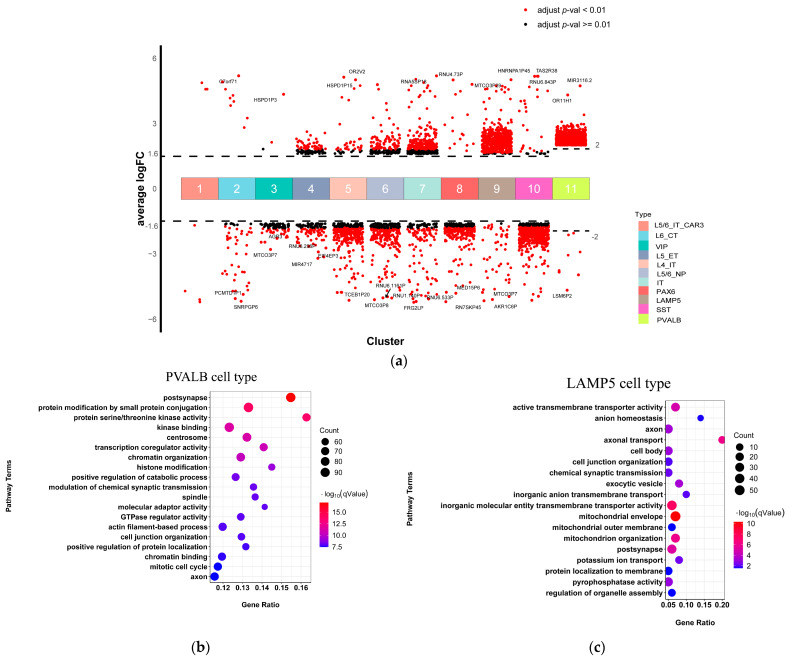

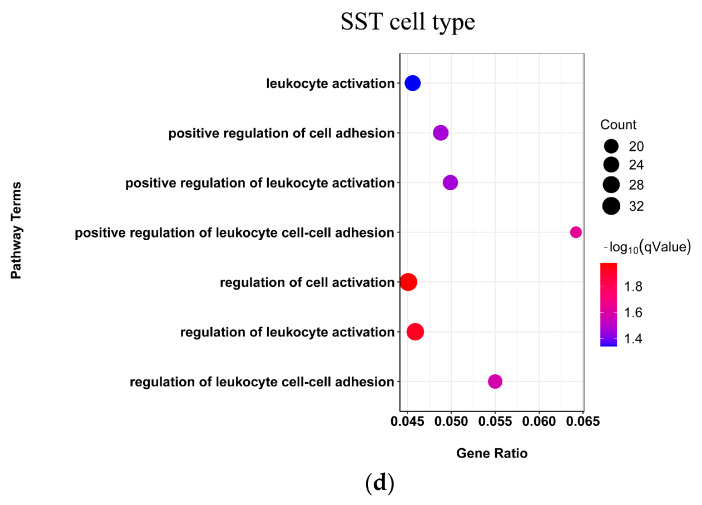
Neuronal type-special DEGs and related pathways of scRNA-seq. (**a**) Differential gene expression analysis showing up- and down-regulated genes across all 11 neuronal clusters. An adjusted *p*-value < 0.01 is indicated in red, while an adjusted *p*-value ≥ 0.01 is indicated in black; (**b**–**d**) The top significantly overrepresented GO terms. The x-axis represents the gene ratio. The y-axis indicates the items of GO, the color of dots represents the value of -log (FDR adjusted *p*-value), and the size of dots represents the count of genes.

Next, we investigated the patterns of expression difference of DEGs in each cell type in SCZ. Based on the gene expression profiles of each cell type from the CRBERSORTx groupmode analysis, we compared gene expression profiles from SCZ and control samples for each cell type following CIBERSORTx guidelines [22]. At the threshold of false discovery rate (FDR) of <0.05 and |log2 fold change| > 1.6 (PVALB cell type |log2 fold change| > 1.6), we identified the DEGs for each neuronal cell type (Figure 3a; Table S1). PVALB, SST, and LAMP5 neurons had the largest number of DEGs. We conducted a functional enrichment analysis on DEGs of each cell type, respectively (Figure 3b), to identify SCZ-associated pathways shared across neuronal cell types and those specific to individual cell types. First, we observed that the PVALB neuron was significantly enriched with gene ontology terms related to the modulation of chemical synaptic transmission, synaptic signaling, postsynapse, and dendrite (Figure 3b). We then found that the LAMP5 neuron was significantly enriched with gene ontology terms related to axodendritic transport, chemical synaptic transmission, axon development, anion transmembrane transport, axon, and postsynapse (Figure 3c). Moreover, functional enrichment analysis indicated that the terms chemical synaptic transmission and postsynapse were enriched in DEGs in both PVALB and LAMP5 neurons. This result suggested that both cell types were involved in chemical synaptic transmission dysregulation in schizophrenia. SST cell type was significantly enriched with gene ontology terms related to positive regulation of neutrophil degranulation and positive regulation of leukocyte cell-cell adhesion (Figure 3d). This result suggested that SST cell type was associated with immune function dysregulation in schizophrenia. No significance was observed for any of the other cell types.

### 2.4. Oligodendrocyte and Endothelial Cells Involved in Immune Function Dysregulation in SCZ

**Figure 4:**
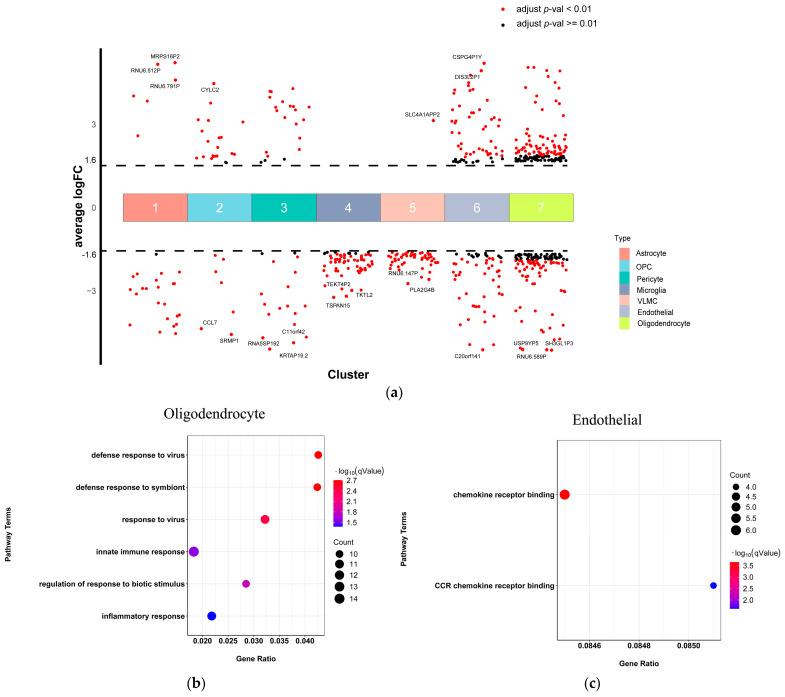
Non-neuronal type-special DEGs and related pathways of scRNA-seq. (**a**) Differential gene expression analysis showing up- and down-regulated genes across all seven non-neuronal clusters. An adjusted *p*-value < 0.01 is indicated in red, while an adjusted *p*-value ≥ 0.01 is indicated in black; (**b**,**c**) The top significantly GO terms. The x-axis represents the gene ratio, the y-axis indicates the items of GO, the color of dots represents the value of −log (FDR adjusted *p*-value), and the size of dots represents the count of genes.

Next, we analyzed the SCZ-associated pathways of each non-neuronal cell type. Under the threshold of false discovery rate (FDR) of <0.05 and |log2 fold change| > 1.5, we identified the DEGs for each non-neuronal cell type and visualized them by volcano plots (Figure 4a; Table S2). Oligodendrocyte and endothelial cells exhibited the top two numbers of DEGs. To identify SCZ-associated pathways that were shared across non-neuronal cell types and those that were specific for individual cell types, we conducted a functional enrichment analysis on the DEGs of each cell type. Oligodendrocyte cells were significantly enriched with gene ontology terms related to the defense response to a virus, pattern recognition receptor signaling pathway, and inflammatory response (Figure 4b). Endothelial cells were significantly enriched with gene ontology terms related to chemokine receptor binding (Figure 4c). This result suggested that both oligodendrocyte and endothelial cells were associated with immune function dysregulation in schizophrenia. No significant enrichment was observed for any other cell types.

### 2.5. Bulk RNA-Seq DEGs Expression Changes at the Cellular Layer

**Figure 5:**
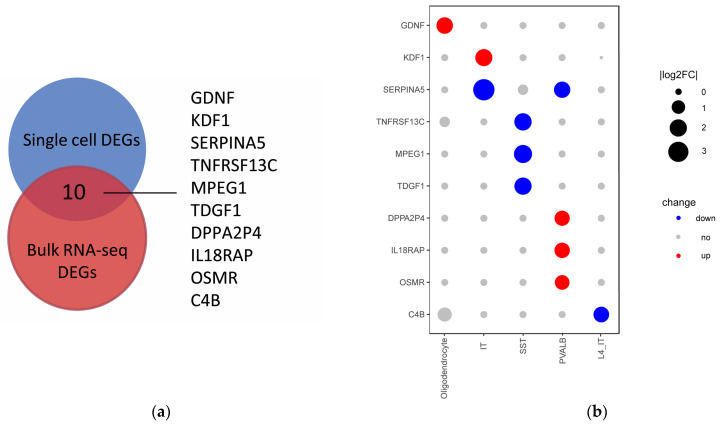
DEGs overlapped between bulk and sing cell RNA-seq. (**a**) Venn diagram of bulk and sing cell RNA-seq DEGs; (**b**) Dot plots showing the relative expression change of specific genes across different cell types. The size indicates the Log2FC values (SCZ/control), the color red indicates upregulated, blue indicates downregulated, and grey indicates no change.

To explore specific changes in the expression of molecules in each cell type in the brain DLPFC region, we evaluated the changes in the expression of bulk RNA-seq DEGs in the cell layer. There are ten genes (TNFRSF13C, MPEG1, OSMR, KDF1, GDNF, TDGF1, C4B, SERPINA5, DPPA2P4, and IL18RAP) overlapped between bulk RNA-seq and scRNA-seq DEGs (Figure 5a). Then, we attempted to explore the cellular level expression changes of these genes. After filtration of low-quality genes, we finally gained expression changes of ten DEGs in five cell types. As shown in Figure 5b, most of the DEGs exhibited expression significantly dysregulated in SST and PVALB cell types. For the SST cell type, TNFRSFQ13C and MPEG1, which were related to immune response [23,24], were significantly downregulated. TDGF1, known for playing a crucial role in human brain development [25], was also downregulated in SST cell types. For the PVALB cell type, SERPINA5, which is implicated in synaptic plasticity and memory formation [26] and was downregulated in bulk RNA-seq, and was also downregulated in IT, PVALB cell types. IL18RAP, known as a proinflammatory cytokine involved in inducing cell-mediated immunity [27], was upregulated in PVALB cell types. In addition, GDNF, which was reported that the endogenous dosage correlates with clinical severity in schizophrenia [28], was upregulated in oligodendrocytes. C4B, which is involved in synaptic phagocytosis [29], was downregulated in the L4_IT cell type. Taken together, we found that the expression changes of several SCZ-related genes are cell-type-specific.

### 2.6. The Relationships between Each Cell Type Identifying Two Major Modules Underlying SCZ

**Figure 6:**
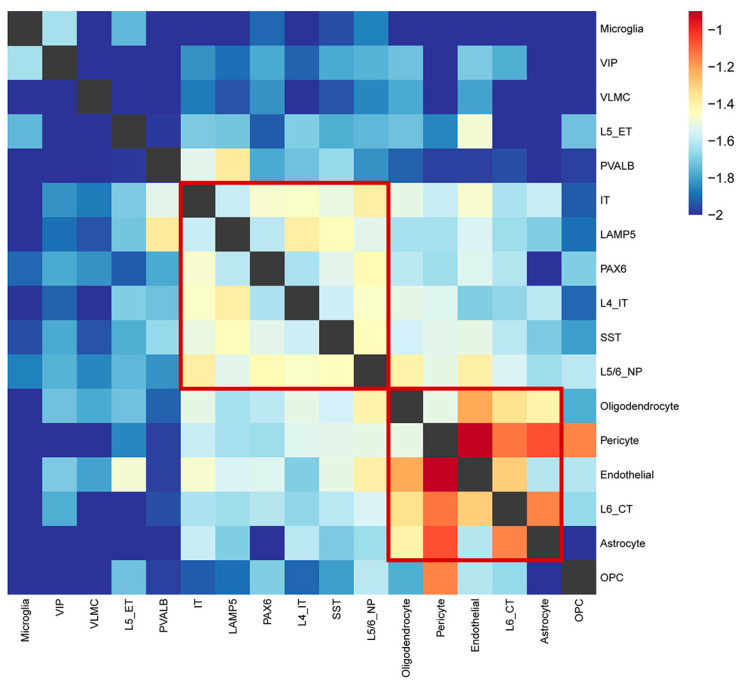
Heatmap showing neuronal and non-neuronal cell types grouped based on Jaccard similarity of the DEGs. Rows and columns correspond to cell types, and the intersection represents the Jaccard similarity between the two cell types. The red box indicates the cell types that converge to a module.

To explore the potential correlation between each cell type underlying SCZ, we plotted the Jaccard similarity heatmap of 17 cell types based on the similarity of the DEGs (Figure 6). There were few DEGs of L5/6_IT_CAR3 cell types, so we omitted this cell type. We observed two correlative modules. One of the modules included three types of excitatory glutamatergic neurons (IT, L4_IT, and L5/6_NP) and three types of inhibitory GABAergic neurons (LAMP5, SST, and PAX6). The other module included four non-neuronal cell types (astrocyte, endothelial, oligodendrocyte, pericyte) and an excitatory glutamatergic neuron (L6_CT). Overall, it suggested that some cell types might converge to a module to affect the SCZ.

## 3. Discussion

Schizophrenia is a complex psychiatric disorder affected by various cell types in the brain. However, how individual cell types are affected by schizophrenia and how each cell type can contribute to schizophrenia occurrence is not completely clear. We sought to address the question by focusing on the integrative analyses of the DLPFC bulk RNA-seq data and scRNA-seq data converted by the deconvolution algorithm CIBERSORTx. We could figure out how changes in the transcriptome of the schizophrenia DLPFC tissue were distributed across multiple cell types.

In this study, we presented the single-cell research of multiple cell types in the DLPFC of schizophrenia patients using CIBERSORTx and elucidated the cell type fraction changes and SCZ-related alterations in gene expression in each cell type at the single-cell level. First, after deconvolution of the bulk RNA-seq data with CIBERSORTx, the proportional distribution of the 19 cell types in SCZ and normal tissues showed a different trend, astrocyte, pericyte, and PAX6 cells were found to have a higher proportion in SCZ patients, while the L5/6_IT_CAR3 cells and LAMP5 cells are the exact opposite. Firstly, astrocyte cells can critically affect key neurodevelopmental and homeostatic processes pertaining to schizophrenia pathogenesis [30]. Our result is consistent with the previous study, which noted postmortem histological study identified that the number of astrocytes was increased in the brain of patients with schizophrenia [31], and the increased density of S100β^+^ astrocytes was found in patients with paranoid schizophrenia [32]. Secondly, with the pericyte cell type, known as one of the neurovascular unit elements, brain imaging in SCZ patients reveals vascular dysfunction in the PFC region using fMRI [33]. Lastly, for GABAergic neurons, PAX6 and LAMP5, and glutamatergic neurons, L5/6_IT_CAR3, which can establish inhibitory and excitatory synapses, the changes in their proportion may impact on alteration of the GABAergic and glutamatergic balance [34].

Next, although bulk RNA-seq [35] has revealed some genes and pathways associated with SCZ, it could not resolve the cell type-specific pathology underlying SCZ. Using single-cell deconvolution analysis of the SCZ DLPFC region greatly increases the resolution of gene expression changes. Thus, we discovered that SCZ is characterized by the dysregulation of thousands of genes with up-/downregulation in specific cell types. In total, by summing up dysregulated genes across all cell types, ~4607 and 528 DEGs for neurons and non-neurons were found, respectively, and each cell type has cell-special DEGs. This suggested that the contribution of each cell type to SCZ occurrence differs in severity. On the one hand, in neurons, we reported that three GABAergic neurons: PVALB, LAMP5, and SST cell types exhibited the largest transcriptomic effect in SCZ. GABAergic neurons play an essential role in regulating neurotransmission and maintaining a fine-tuned excitation-inhibition balance in the brain [36]. Firstly, we found that the PVALB cell type exhibited abnormality in synaptic signaling and chemical synaptic transmission. The result is consistent with the glutamate hypothesis of schizophrenia states, which noted the PVALB neurons mediated glutamate neurotransmission to attribute to the development of SCZ [37]. Secondly, LAMP5 neurons were among the cell types most affected by SCZ based on the changes in gene expression and cell-type proportion. LAMP5 interneurons exhibited abnormality in ATP metabolic process, NADH dehydrogenase complex assembly, chemical synaptic transmission, axo-dendritic transport, and axon development. This result suggested that LAMP5 neurons not only take part in GABAergic neurons’ common neurotransmission function but also play an important role in energy metabolism in SCZ. Lastly, the SST neurons exhibited abnormalities in leukocyte cell-cell adhesion, leukocyte activation, and T cell activation. In some mouse models, SST neurons have been shown to be associated with immune and neuroinflammation [38]. On the other hand, in non-neurons, we reported that oligodendrocytes exhibit the largest transcriptomic effect in SCZ. Here, oligodendrocytes exhibited abnormalities in their innate immune response and inflammatory response. Similarly, various studies also support that oligodendrocytes are directly involved in inflammation and immune modulation in CNS disease [39,40].

In addition, one of the major findings in our study is the discovery that the DEGs from bulk RNA-seq display different cellular-level changes. For example, the SERPINA5 gene exhibited a dramatic decrease in expression across two neuron cell types, IT and PVALB. The SERPINA5 gene also exhibited downregulation in L5/6_NP and IT cell types, although they were not significant enough. Interestingly, SERPINA5 encodes a member of the serine protease inhibitor family of proteins, which has been implicated in synaptic plasticity and memory formation [41]. Angela M. Crist et al. reported that histologic and biochemical analyses suggested SERPINA5 expression dysregulation is associated with Alzheimer’s disease [26]. SCZ and AD are two severe brain disorders that share considerable comorbidities in both clinical and genetic contexts [42]. Thus, the genetic influence of SERPINA5 on SCZ is also worth exploring.

Furthermore, considering that SCZ is caused by the cooperation of multiple cell types, we used Jaccard distance to identify two major cell type modules. The cell types converged to the same module and exhibited similar coordinated transcriptomic changes. Firstly, three excitatory glutamatergic neurons (IT, L4_IT, and L5/6_NP) and three inhibitory GABAergic neurons (LAMP5, SST, and PAX6) clustered together according to changes in gene expression. This result suggested that two major neurons exist in coordinated shifts, inconsistent with the previous observation that glutamatergic and GABAergic neurons lost connection [43] in brain regions associated with SCZ. Secondly, the expression pattern of non-neuronal cell types was different from neuronal cell types. For example, the cross-talk between astrocytes and oligodendrocytes is important for glial development, triggering disease onset and progression, as well as stimulating regeneration and repair [44]. We also observed that glutamatergic neurons (L6_CT) exhibit a similar expression change to non-neuronal cell types. It is reported that inflammatory cytokines such as IL-1β released from glia may facilitate signal transmission through its coupling to neuronal glutamate receptors [45]. This bidirectional neuron-glial signaling plays a key role in glial activation and cytokine production [46]. Overall, such modules of SCZ-related cell types can reveal the cooperation of multiple cell types, which may influence the occurrence of SCZ.

Taken together, although our current study has some limitations, we identify large-scale changes in the neuronal and non-neuronal transcriptomes of SCZ patients using CIBERSORTx, where some cell types displayed proportion changes and a dramatic SCZ-related cell-specific dysregulation of gene expression. Moreover, by integrating analyses of the DLPFC bulk RNA-seq and scRNA-seq data, we reveal that the DEGs from bulk RNA-seq display different changes at the cellular level. In addition, we discovered that different subtypes of neurons might exhibit coherent SCZ-related transcriptomic changes. Our results suggest that SCZ therapies should take into consideration the genes and cell types heterogeneity. Thus, these findings strengthen our understanding of SCZ and can improve current therapeutic strategies for this disorder.

## 4. Materials and Methods

### 4.1. Data Collection

We obtained the single-cell transcriptome data of 6468 cells with normal adult middle temporal gyrus (MTG) and anterior cingulate cortex (ACC) from the ALLEN BRAIN MAP [16,17] (https://portal.brain-map.org/atlases-and-data/rnaseq/human-multiple-cortical-areas-smart-seq, accessed on 24 February 2022). The human dorsolateral prefrontal cortex (DLPFC) RNA-seq data in this study were collected from BrainSeq Phase 2 [18] (http://eqtl.brainseq.org/phase2, accessed on 24 February 2022), consisting of 138 SCZ cases and 251 control samples over 18 years of age.

### 4.2. Impute Cell Fractions with CIBERSORTx

To characterize the abundance of 19 cell types based on the RNA-seq data in DLPFC tissues, we applied CIBERSORTx web tool, a machine learning method that infers cell-type-specific gene expression profiles without physical cell isolation. We first prepared and uploaded the single-cell expression matrix according to the instructions with CIBERSORTx. All parameters used the default values suggested by the tool developers. Then we ran “CIBERSORTx” and obtained a signature matrix of 19 cell types from scRNA-seq data.

Next, we loaded the datasets of SCZ patients and healthy subjects’ samples into CIBERSORTx according to the instructions. After inputting the cell type signature matrix, we acquired the relative proportions of 19 neuronal and non-neuronal cells in each sample with a *p*-value corresponding to the confidence of the results for the deconvolution. Because the scRNA-seq data was derived from Smart-seq2 platform [47], we selected “B-mode” for batch correction and 1000 permutation tests. Other parameters used the default values.

### 4.3. Bulk RNA-Seq Differentially Expressed Gene Analysis

We analyzed the bulk RNA-Seq data of 138 SCZ cases and 251 control samples obtained from BrainSeq Phase 2. The analysis was performed using package “DESeq-2” [19], “limma” [20], and “edgeR” [21]. Setting the cut-off criteria as |log2 fold change| > 0.5 and FDR < 0.05, we identified 108 differentially expressed genes (DEGs) that reached the significance threshold in all three methods.

### 4.4. Impute Cell-Type-Specific Gene Expression with CIBERSORTx

To impute cell type-specific gene expression, we performed CIBERSORTx group mode on SCZ and normal classes from BrainSeq Phase 2 separately. Both expression profiles produced by the CIBERSORTx group mode had been filtered out using a threshold to eliminate unreliably estimated genes for each cell type. The steps of the CIBERSORTx group mode included: (1) For each cell phenotype, CIBERSORTx set genes with low average expression (<0.75 transcripts per cell) in log2 space to 0 as a quality control filter. (2) To further reduce confounding noise, CIBERSORTx filtered genes based on their geometric coefficient of variation. Then using filtered gene expression profiles, we identified statistically significant differentially expressed genes of each cell type using the R script provided by the guideline. The cut-off criteria were false discovery rate (FDR) of <0.05 and |log2 fold change| >1.5.

### 4.5. Gene Ontology (GO) Analysis

We performed a GO enrichment analysis of the DEGs of each cell type by Metascape [48]. Functional enrichment was performed in three GO categories: biological process, molecular function, and cellular component. Terms with *p* < 0.05, a minimum count of 3, and an enrichment factor of >1.5 (the enrichment factor was defined as the observed count’s ratio to the count expected by chance) were collected and grouped into clusters based on their membership similarities. Furthermore, *p*-values were calculated based on the cumulative hypergeometric distribution. The *p*-values of the hypergeometric tests were adjusted for multiple testing by the Benjamin–Hochberg method.

### 4.6. Jaccard Similarity between Each Cell Types

To explore the relationships between each cell type underlying the SCZ, we estimated the Jaccard similarity on the DEGs of each cell type.

$$\mathrm{Jaccard}\;\mathrm{similarity}=\frac{N_{\mathrm{both}\;\mathrm{cell}\;\mathrm{types}}}{N_{\mathrm{either}\;\mathrm{cell}\;\mathrm{type}}}$$

For the visualization, we clustered Jaccard similarity using hierarchical clustering (R functions hclust with parameter method = “ward.D”).

## Figures and Tables

**Table 1:** Marker genes of each cell type in the signature matrix.

Board Class	Subtype	Top Marker Genes
GABAergic (inhibitory)	LAMP5	LAMP5, GGT8P, NDNF, DUSP4, CA13, SFTA3, C1QL2, ANKRD20A11P
	SST	SST, MTHFD2P6, MAFB, ISOC1, KLHL14, AHR, NPY
	VIP	VIP, TOX2, ZNF322P1, CBLN1, CXCL14, PPAPDC1A, ADARB2, ADAM33, CHRNA2, KCNJ2, SSTR1, PRSS8
	PAX6	PAX6, GRIP2, CA4, SCGN, NABP1
	PVALB	PVALB, FAM150B, CNTNAP3P2, WFDC2, STON2, LHX6, GLP1R, SCUBE3, TAC1, MFI2, C8ORF4
Glutamatergic (excitatory)	L4_ IT	RORB, GRIK1, RPS3P6, HLHE22, ACNG5, CDC168, AIM2, ASCL1
	L5_ET	FEZF2, SCN7A, ONECUT1, DCN, MORN2
	L5/6_NP	FEZF2, MYBPHL, CYP26B1, DYRK2, CABP7, RSAD2
	L5/6_IT_CAR3	THEMIS, GPR21, C6ORF48, THTPA, IL7R
	L6_CT	FEZF2, FAM95C, ANKRD20A1, CPZ, ETV4, VWA2
	L6b	FEZF2, KRT17, TBC1D26, SLITRK6, P4HA3, TBCC
	IT	LINC00507, RPL9P17, RORB, RPL31P31, LCN15, THEMIS, LINC00343, SNHG7, SEMA6D, PRSS12, LINC01474, LINC01202
Non-neuron	Astrocyte	FGFR3, ETNPPL, MT1G, FOS
	Endothelial	CLDN5
	Microglia	C1QC
	Oligodendrocyte	OPALIN, MOBP, COL18A1
	OPC	MYT1
	Pericyte	MUSTN1
	VLMC	CYP1B1

## Data Availability

All data analyzed in this study are curated from the public domain.

## Supplementary Materials

The following supporting information can be downloaded at: https://www.mdpi.com/article/10.3390/ijms231911581/s1. The data and R codes involved in this study are available on GitHub at (https://github.com/CWENXIANG/SCZ-scRNA-seq-analysis, accessed on 20 September 2022).
